# Coagulation, Microenvironment and Liver Fibrosis

**DOI:** 10.3390/cells7080085

**Published:** 2018-07-24

**Authors:** Niccolò Bitto, Eleonora Liguori, Vincenzo La Mura

**Affiliations:** 1 Medicina Interna, Istituto di Ricovero e Cura a Carattere Scientifico (IRCCS) San Donato, Università Degli Studi di Milano, 20097 San Donato Milanese (MI), Italy; nicobitto@gmail.com (N.B.); eleonora.liguori1982@gmail.com (E.L.); 2 Fondazione IRCCS Ca’ Granda, Ospedale Maggiore Policlinico, UOC Medicina Generale-Emostasi e Trombosi, 20122 Milano, Italy; 3 Dipartimento di Scienze biomediche per la Salute, Università degli Studi di Milano, 20122 Milano, Italy; 4 A. M. and A. Migliavacca per lo studio delle Malattie del Fegato, 20122 Milano, Italy

**Keywords:** thrombin, protease-activated receptors, endothelial dysfunction, von Willebrand factor, hepatitis, cirrhosis, anticoagulation

## Abstract

Fibrosis is the main consequence of any kind of chronic liver damage. Coagulation and thrombin generation are crucial in the physiological response to tissue injury; however, the inappropriate and uncontrolled activation of coagulation cascade may lead to fibrosis development due to the involvement of several cellular types and biochemical pathways in response to thrombin generation. In the liver, hepatic stellate cells and sinusoidal endothelial cells orchestrate fibrogenic response to chronic damage. Thrombin interacts with these cytotypes mainly through protease-activated receptors (PARs), which are expressed by endothelium, platelets and hepatic stellate cells. This review focuses on the impact of coagulation in liver fibrogenesis, describes receptors and pathways involved and explores the potential antifibrotic properties of drugs active in hemostasis in studies with cells, animal models of liver damage and humans.

## 1. Introduction

Fibrogenesis is a complex biochemical process that represents the hallmark of damage for the most common chronic diseases of the liver. The activation of hepatic stellate cells (HSC) is the key pathogenic mechanism for the initiation, progression, and regression of liver fibrosis. Several studies have gone into more depth on the complex and tightly regulated cross talk at the level of hepatic microcirculation owing to sinusoidal endothelial cells (SEC), Kuppfer cells (KC), and hepatocytes with HSC. This underlines the participation of several hepatic cellular types in fibrogenesis. Our manuscript offers an overview on the pathogenic role played by coagulation and thrombin generation in this complex cellular cross talk by considering fibrosis a wound healing process secondary to micro-thrombi in small hepatic and portal venules, sinusoidal ischemic injury and hepatocyte injury. In addition, thrombin may participate in fibrogenesis by interaction with HSC via protease-activated receptors (PAR-1 and PAR-4), promoting a myo-fibroblast phenotype, fibronectin fibril assembly, and may act as a chemoattractant for inflammatory cells. Altogether, these observations suggest that drugs interfering with the coagulation process have potential as antifibrotic drugs at any stage of chronic liver disease. The in vitro and in vivo studies on these aspects are the main focus of the review.

## 2. Coagulation in Fibrosis and Disease Progression

### 2.1. Hepatic Stellate Cells, Endothelium and Fibrosis: Role of PARs

**Figure 1:**
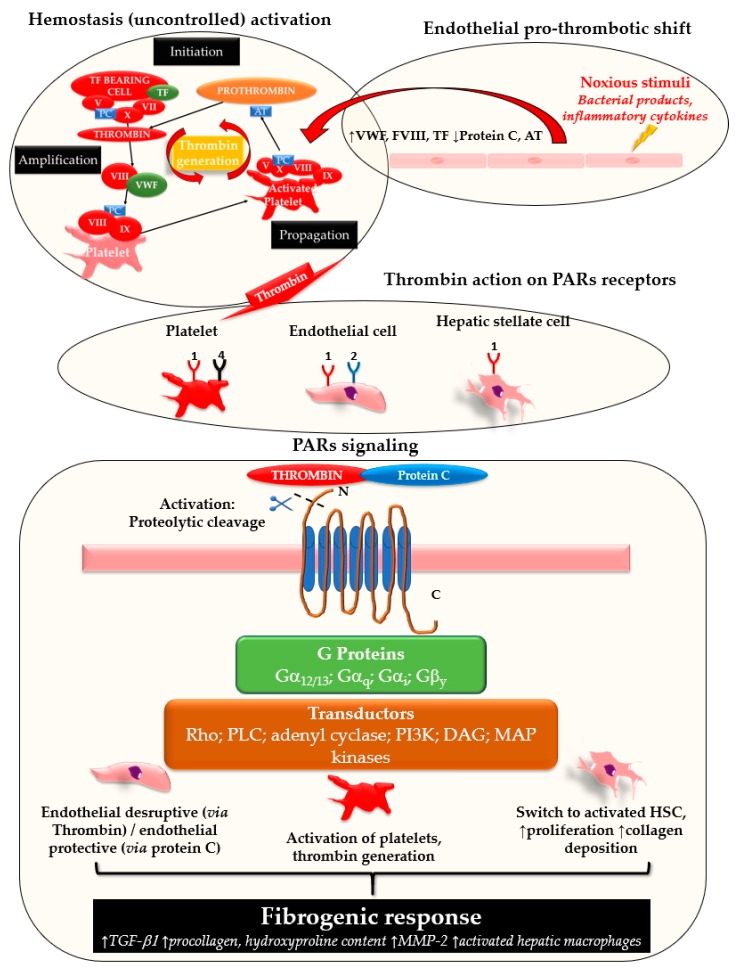
Schematic representation of PAR signaling.

During coagulation, the conversion of fibrinogen into fibrin is a key reaction catalyzed by thrombin, a serine protease which is generated on the surface of activated platelets in response to vascular or tissue injury [1]. Thrombin generation is a tightly regulated process, as it is the expression of the delicate balance between pro-coagulant and anti-coagulant factors. Besides its hemostatic function, thrombin orchestrates cell recruitment in response to any kind of tissue injury and activates endothelium [2,3,4]. Its interaction with inflammatory and mesenchymal cells is part of the wound healing process, in which hemostasis precedes and initiates tissue repair by fibrin deposition [5]. In 1991, the discovery of protease activated receptors (PARs) clarified the biological pathway of thrombin [6]. PARs are a family of receptors with proteolytic activity, which mediate thrombin (PAR 1, 3, 4)- or tryptase (PAR 2, 4)-induced cellular response. PARs are G-protein-coupled receptors and are activated by irreversible proteolytic cleavage of their N-terminal domain. They are expressed by several cellular types involved in fine regulation of vascular homeostasis and their signaling pathways are complex, as they are potentially coupled to G-proteins with different functions (Figure 1). As a result, they interact with a plethora of signaling transducers (e.g., Rho/Rho-kinase, c-Jun N terminal kinase, IP3, PI3K, JAK-STAT), with consequent pleiotropic effects [7,8]. Endothelium (via PAR1, PAR2) and platelets (PAR1, 4), are the main cells involved in the regulation of vasomotor function and hemostasis exerted by PARs [9]. At low concentrations, thrombin may induce a barrier protective response by endothelium, this effect is mediated by PAR-1 [10]. On the contrary, at high concentrations, thrombin induces a pro-inflammatory, pro-hemostatic and contracting phenotype of endothelium, as it increases the expression of TF, plasminogen activator inhibitor-1 expression (PAI-1), pro-inflammatory cytokines (IL6, IL8) and endothelin-1, among others [7]. This bi-modal effect of thrombin suggests that a disrupted regulation of thrombin generation, as occurs in pro-coagulant conditions, may overcome its physiological interaction with the endothelium and may induce significant tissue injury. Alongside endothelium, platelets are activated by PAR-1 and PAR-4, and inhibition of these receptors is a potent anti-platelet mechanism, confirming the important role played by these receptors on platelet function [11,12]. Thrombin is produced on the surface of activated platelets and its interaction with PARs may initiate and maintain the hemostatic process, leading to thrombus formation when anticoagulant factors are not able to counterbalance this process [1,7]. In recent years, the transcription factor Kruppel-like factor 2 (KFL2) has been recognized as a key regulator of endothelium homeostasis in response to inflammatory stimuli (e.g., tumor necrosis factor, TNFα, and interleukin 1) and hemodynamic forces like laminar shear stress [13,14]. Interestingly, Marrone et al. demonstrated that KLF2 overexpression in SEC and HSC proceeding from cirrhotic rats reduces HSC activation and ameliorates paracrine cross-talk with SEC [15,16]. This is in line with the reduction of fibrosis and portal pressure observed in animal studies in association with KFL2 expression [17]. In 2005, Li et al. demonstrated that KLF2 induction blunts the pro-inflammatory, pro-hemostatic transcriptional response of the endothelium exposed to noxious stimuli (e.g., TNFα), as it reduces tissue factor, Von Willebrand Factor (VWF) and PAI-1 [18]. Interestingly, ADAMTS-13, a metalloproteinase which regulates the pro-hemostatic function of VWF by its cleavage, is produced by HSC in physiological conditions and its activity declines alongside liver dysfunction [19,20]. Absolute deficiency of ADAMTS-13 leads to diffuse microvascular occlusion due to high molecular weight VWF multimers which promote platelet aggregation and microthrombi formation; therefore, low levels of ADAMTS-13 are of increasing interest in thrombotic-microangiopathies and those clinical conditions like sepsis in which liver failure, as well as other organ dysfunctions are frequently observed [21,22]. All these observations emphasize the role of HSCs-SEC interaction to maintain an anti-thrombotic phenotype at sinusoidal level in physiology, with a potential protective role of KFL2 due to its control of VWF and platelet aggregation. Moreover, KFL2 may also have a direct role in the direct control of hemostasis by the endothelium, since it inhibits PAR-1 expression on endothelial cells. This shows a direct link between KLF-2-induced regulation of endothelial physiology and the biological response of this cytotype to thrombin. Whereas in vascular medicine studies on PARs focus on platelets and endothelium, PARs expression by HSCs is central in liver fibrogenesis [17]. The progression of any chronic liver disease is characterized by the acquisition of a contractile and pro-fibrogenic phenotype by HSCs, along with an imbalance between vasoconstrictors and vasodilators produced by SECs. As a consequence, the liver parenchyma is distorted by the development of interstitial fibrosis and the constriction of sinusoids which, in turn, lead to the increase of portal pressure owing to the mechanical and functional increase of liver resistance to the portal blood flow [23]. Several studies have explored thrombin-PARs interaction on HSC in the process of liver fibrogenesis. They are summarized in Table 1. The action of thrombin on PARs (mainly PAR-1) induces fibrogenic response in the liver by reprogramming HSCs with the induction of a pro-fibrotic, activated phenotype [24,25,26]. Incremental doses of thrombin progressively transform HSCs into myofibroblasts, with increase of αSMA, pro-collagen, TGFβ-1 and other key cellular signals which are crucial in the wound healing response [24]. The uncontrolled persistence of a thrombin-related signaling through PARs, due to a pro-hemostatic milieu, is considered the main mechanism that binds hemostasis and fibrosis [27]. In line with this theory, experimental inhibition of PARs prevents the fibrogenic response of HSCs and the progression of fibrosis as demonstrated in pre-clinical studies with animal models of liver disease and cell cultures [26,28]. In addition to PAR-1, PAR-2 showed similar pro-fibrotic effects by inducing HSC contraction, collagen production and MMP-2 expression, this last promoting liver fibrosis due to extracellular matrix remodeling [25,29,30,31]. Furthermore, studies with PAR^−/−^ transgenic mice confirmed the importance of this receptor in several models of liver fibrosis (xenobiotics, carbon-tetrachloride, CCL_4_, and thioacetamide, TAA) [30,32,33] and, recently, even in a model fatty liver disease [34]. To our knowledge, just one study explored PAR-1 genotype and liver fibrosis in patients with chronic HCV infection. In this biopsy-proven study, a particular PAR-1 polymorphism (1426 C/T) correlated with increased liver fibrosis, thus confirming the above-mentioned results from pre-clinical studies [35]. Alongside PARs, tissue factor (TF) has been often investigated in liver fibrosis, since TF is a potent activator of hemostasis via factor VII (FVII) [36]. Interestingly, transgenic mice lacking of TF show a reduced rate of fibrosis development after exposure to various chronic damage stimuli, thus confirming a potential connection between the pro-hemostatic role of TF and liver fibrosis [31,34]. Recently, Ratou et al., in a study with mice after bile duct ligation (an animal model of liver fibrosis), demonstrated an increase of thrombin-antithrombin complexes, which are biomarkers of a pro-coagulant condition. This increase was prevented in mice lacking in TF. However, this anti-coagulant phenotype was not associated with a significant reduction of fibrosis [37], in contrast to other studies [30]. 

In summary, hemostasis may drive a pro-fibrotic HSC phenotype via PARs. The cellular cross talk between HSCs and SECs and the expression of KLF2 may somehow reduce the fibrogenic process associated with a pro-coagulant imbalance under chronic conditions of liver damage. 

### 2.2. Parenchymal Extinction: From Clot Generation to Liver Damage

An important step in the knowledge of coagulation as a mechanism of liver damage was the study by Wanless et al., who conducted a histological analysis by comparing 61 cirrhotic livers of any etiology removed at the time of transplantation with 24 livers from autopsy of normal subjects as controls [39]. The main purpose of the study was to confirm the previous observation that fibrosis co-localizes with vascular lesions of the hepatic venous system [40]. First, they distinguished origin (hepatic or portal), caliber (small, medium or large) and size (% of luminal narrowing) of vascular lesions. Second, they graded fibrosis with description by optical microscope and defined focal parenchymal extinction as a region of parenchymal loss filled by fibrosis. Hepatic and portal vein intimal fibrosis, highly suggestive of previous occlusion, were respectively evident in 70% and 36% of livers. In morphometric data on 534 hepatic veins pooled from 10 livers, hepatic vein occlusions were frequent in small veins and co-localized with a greater extent of fibrosis. The existence of a “post thrombotic syndrome” was also inferred by patchy distribution of fibrotic areas, multiple layers of fibrosis and the severe occlusion of the smallest veins. In another study, the same group analyzed 13 autopsy livers with congestive fibrosis, with another 12 livers as controls [41]. In this model, venous stasis was associated with thrombosis of sinusoids and terminal hepatic venules, with formation of fibrotic septa and sinusoidal fibrotic thickening. These changes were associated with the extension of thrombosis to larger veins, necrosis and parenchymal loss. Recently, Simonetto et al. confirmed these results in an animal model of congestive hepatopathy (partial inferior vena cava ligation), showing hepatic sinusoidal thrombosis with increase of liver stiffness and portal pressure [42]. Interestingly, fibrosis was accompanied by minimal inflammation, whereas mechanical forces seemed to prevail with stretch-induced fibronectin-fibrils assembly. Of note, tissue factor pathway inhibitor or warfarin treatment blunted sinusoidal thrombosis and fibrosis deposition, confirming the existence of a hemostasis-driven fibrogenesis in this model of liver congestion. They also analyzed liver specimens of patients with congenital heart failure due to chronic myocardial dysfunction or Fontan cardiac surgery, which is a set of surgical techniques causing venous hypertension after deriving the systemic venous flow directly into the pulmonary artery. In these patients, immunochemistry analysis revealed fibrin deposition within sinusoids, confirming the association between microthrombosis and fibrosis. The lack of inflammation in this study is apparently in line with the pre-clinical study by Cerini et al. who demonstrated a minimal anti-inflammatory effect of heparin, alongside a potent anti-fibrotic impact prevalently due to the anticoagulant properties of the drug. These results are in line with a recent work by Miyao et al., who demonstrated in a mice model of non-alcoholic fatty liver disease that sinusoidal endothelial injury may precede the activation of Kupffer cells, HSC, inflammation and fibrosis [43]. Therefore, despite inflammation being a cardinal element in development of a biological response to every kind of noxious stimuli, its link with hemostasis probably cannot explain alone the consequent fibrogenic response. Recent evidence, elsewhere reviewed by De Ridder et al. [44], focuses on the precise site of thrombin generation, identified in the intravascular or the interstitial anatomical space. Whereas intravascular activation is easily understood and studied in micro and macrovascular medicine, thrombin activity in the interstitial space is intriguing and often neglected [44]. However, liver fibrosis is, by definition, an interstitial process, and it is conceivable that thrombin exerts an important and complex action on fibrogenic response, for example by activating pro- [45] and anti-fibrotic [46]. metalloproteinases present in the extracellular matrix. The exact link, if it exists, between the intravascular and interstitial generation of thrombin during chronic hepatitis is certainly an open question, which is even more of interest for hepatologists, as recent studies have shown that anti-coagulation per se may favorably impact the natural history of cirrhosis [47,48]. 

In conclusion, parenchymal extinction theory represents the bridge between pre-clinical studies demonstrating a role of hemostasis on liver fibrogenesis and the pathological observations of liver parenchymal loss due to vascular occlusion, progressive necrosis and fibrosis replacement in humans. However, as thrombin explains its action also in the interstitium, further studies are warranted to confirm and define precisely the weight of microvascular and interstitial changes due to the activation of thrombin as consequence of a chronic liver damage. 

### 2.3. Procoagulant Imbalance and Disease Progression: Clinical Observations

#### 2.3.1. Common Inherited Pro-Hemostatic Genotype and Risk of Fibrosis Development

Unprovoked venous-thromboembolic events are often linked with pro-hemostatic mutations of clotting factors. FV Leiden and FII G20210 mutations are associated with thrombotic events in the general population, with relative common frequency (0.4–5% and 3%, respectively) [49,50,51]. FV Leiden missense mutation (ArG506Gln) leads to an intrinsic resistance to the anticoagulant action of protein C, whereas FII G20210 increases prothrombin levels and inhibits fibrinolysis by the reduction of the thrombin-activatable-fibrinolysis-inhibitor [52,53]. The potential impact on fibrosis development of a constitutional pro-thrombotic imbalance has been hypothesized and explored by several authors. In 2003 Wright et al. conducted a retrospective, biopsy-proven study aimed to describe the degree of association between the most common thrombophilic factors and the severity of liver fibrosis. In this study, FV Leiden, but not FII G20210 mutation was associated with accelerated fibrosis and cirrhosis development in patients with HCV infection [54]. In contrast, Maharshak et al. demonstrated an association between faster fibrosis and FII G20210 mutation with no evidence for FV Leiden [55]. These divergent results resemble subsequent data showing a potential [54,55,56,57,58] or doubtful impact of thrombophilia on the risk of liver fibrosis development [57,59,60]. A recent retrospective population study on 1055 patients demonstrated an association between FV Leiden and FII G20210 mutation and a significant increase in liver stiffness, which is a widely used non-invasive marker of liver fibrosis [56]. Moreover, in this study, non-0 blood group showed the highest liver stiffness in patients with pro-hemostatic mutations. These data are in line with a previous observation of an association of non-0 blood group and fibrosis severity in HCV-infected patients [61,62]. Interestingly, AB0 blood group is a major determinant of VWF and factor VIII (FVIII) levels in normal subjects, both potent pro-hemostatic factors, and non-0 blood group has been associated with increased levels of VWF and FVIII with increased risk of venous-thrombo-embolism [63,64]. In summary, a pro-hemostatic genotype may have a role in the development of fibrosis. However, evidence is limited to observational studies. The clinical question if thrombophilic inherited mutations may identify clusters of patients with high risk of fibrosis progression is appealing. Thus, it advocates proof of concept studies to clarify the impact and the magnitude of these mutations on fibrosis development. 

#### 2.3.2. Hemostatic Balance in Advanced Liver Disease 

Every stage of liver disease results in a different degree of change in the hemostatic balance [65]. For years, the alteration of conventional coagulation tests (e.g., prothrombin time, partial activated thromboplastin time, bleeding time) disguised the coagulopathy of liver disease under a bleeding mask, represented by the assumption of spontaneous bleeding among patients with cirrhosis, the final grade of any chronic liver disease [66]. This is true in terms of spontaneous gastro-intestinal bleeding, but today we know that this is a consequence of portal hypertension and not of a disease-related reduction of plasma activating the coagulation cascade [48,67]. Indeed, in cirrhosis, the reduction of liver-dependent pro-hemostatic clotting factors (FII, V, VII, IX, X and XI) is counterbalanced by the reduction of anticoagulant factors and similar contrasting alterations in the fibrinolytic system [65]. As a result, the evaluation of the plasmatic hemostatic balance by the in vitro thrombin generation test, which takes into account both pro- and anti-coagulant factors, showed normal thrombin generation in these patients [68]. Therefore, the first seminal study by Tripodi et al. [68] allowed a shift from the old paradigm of an intrinsic bleeding tendency, to the concept of “re-balanced hemostasis” in patients with chronic liver disease [69,70]. Moreover, the same research group demonstrated a resistance to the action of thrombomodulin, a strong anticoagulant, which parallels disease severity and a progressive pro-coagulant imbalance of clotting factors [71,72]. The hypothesis of a pro-coagulant plasmatic milieu in cirrhosis is intriguing, as thrombotic events are common in this population [73]. Thrombosis of portal and splanchnic venous vessels ranges from 5 to 20%, and the highest rate is observed in the advanced stages of the disease [74,75]. Moreover, retrospective studies have shown that cirrhosis may represent a risk factor for venous-thrombo-embolic events in hospitalized patients [76,77,78]. The increase in FVIII, VWF and the resistance to the action of thrombomodulin due to protein C reduction are the best-described pro-hemostatic features, and worsen along with disease severity [72,79,80]. Interestingly, they were all independently associated with increased portal hypertension and worse prognosis, suggesting a potential impact on the pathogenesis of this clinical condition [81,82,83,84,85]. However, the design of these studies does not allow the uncovering of a cause-effect relationship between fibrosis and pro-hemostatic changes, despite an interesting role for VWF as a noninvasive marker of fibrosis in two studies [86,87]. Nevertheless, a potential impact of coagulation on fibrogenesis and parenchymal extinction is fascinating and is currently under investigation by several research groups. One potential limitation is the lack of a study investigating hemostasis in liver disease far from advanced stages or cirrhosis. Recently, two distinct leading groups in this field have published contrasting evidence on this topic in the clinical setting of non-alcoholic liver disease, which is expected to be the main increasing etiology of cirrhosis in next few years [88,89,90,91]. The recent debate that has risen on this topic [92,93] demonstrates the need of further investigations on the impact of hemostasis even in the earliest stages of any chronic disease of the liver. 

## 3. Anticoagulation as Anti-Fibrotic Strategy

### 3.1. Heparin

In the era of etiological therapies, which will hopefully erase the burden of chronic viral hepatitis [94], powerful antifibrotic drugs are still lacking [95,96]. However, the increasing incidence of metabolic liver disease calls on such therapies, while etiologic treatments for NAFLD/NASH are not yet satisfactory [90]. Several studies have explored antifibrotic proprieties of drugs active on hemostasis (Table 2). Low molecular weight heparins (LMWH) inhibit factor X indirectly via antithrombin, thus lowering thrombin generation [97]. In a histological study in rats exposed to carbon-tetrachloride (CCL_4_), LMWH reduced fibrosis and collagen deposition, while ultrastructural analysis on transmission electron microscope (TEM) showed reduced sinusoidal swelling and less distorted parenchymal architecture [98]. Dalteparin also showed fibrosis reduction in CCL_4_ chronic damage, while increasing hepatic-growth factor and blunting pro-fibrotic expression of TGF-β1 and deactivating HSC (αSMA reduction). Interestingly, no effect on necrosis and inflammation was observed, with unchanged levels of TNF [99]. These results were confirmed in a study by Cerini et al., who explored enoxaparin in different rat-models of liver damage: CCL_4_ (acute/short/long exposure) and TAA exposure [100]. Fibrosis and pro-fibrogenic stimuli were analyzed with histology, immunochemistry and HSC isolation. Additionally, portal pressure and hepatic vascular resistance were analyzed with isolation and perfusion of the liver. Enoxaparin markedly reduced fibrosis, with anti-fibrotic reprogramming of HSC with αSMA and pro-collagen I reduction. Moreover, it also reduced portal pressure without altering hepatic blood flow, thus reducing hepatic resistance in accordance to ohm’s law (pressure = flow x resistance). These results were confirmed in both CCL_4_ and TAA damage induction. Indeed, enoxaparin disclosed antifibrotic effects in chronic but not acute liver damage and this occurred without any anti-inflammatory action. Therefore, this solid biological background allows to promote LMWHs as potential antifibrotic strategy. Along these lines, relevant clinical data derive from the trial by Villa et al. [47] who randomized 70 patients with decompensated cirrhosis to receive, or not, enoxaparin in order to prevent de novo portal vein thrombosis. Surprisingly, the treatment arm prevented de novo portal vein thrombosis without any increase of the bleeding rate, and patients showed better clinical outcomes in term of new decompensating events (mainly ascites development) and survival. When treatment was interrupted, both arms turned to similar rates of clinical events and portal vein thrombosis development. This study was the first randomized clinical trial demonstrating the potential impact of anticoagulant on the natural history of cirrhosis, although as of today, no data exist to conclude that the beneficial effect of anticoagulation was mediated by the antifibrotic properties demonstrated in the above-mentioned pre-clinical studies. 

### 3.2. Oral Anticoagulants: From Vitamin K Antagonists to Direct Oral Anticoagulants (DOACs)

Warfarin is an oral anticoagulant which inhibits the production of clotting factors, thus indirectly abolishing thrombin generation [104]. The laboratory testing of INR (a standardized measure derived from prothrombin time) is specifically designed to monitor the anticoagulant effect of vitamin k antagonists [105,106]. In 2008, Anstee et al. studied the effect of warfarin in mice with prothrombotic mutation of FV Leiden exposed to CCL_4_ [101]. In this animal model, warfarin significantly reduced fibrosis progression and liver hydroxyproline content, while mice carrying FV mutation exhibited fibrosis progression with blunted effect of warfarin. In recent years, DOACs have radically changed management in hemostasis modulation [107]. This class of drugs directly inhibits the action of clotting factors (FX and FII), thus reducing thrombin generation [107]. The oral assumption and the lack of need of laboratory monitoring are progressively prompting the repeal of vit k antagonists in favor of this class of drugs, which is currently used in various thrombotic diseases [108]. In 2012, Kassel et al. studied the effect of argatroban, a direct inhibitor of FII, in LDLr^−/−^ fed with a western diet [102]. Argatroban reduced hepatic mRNA expression of αSMA, COL1A1, PDGFβ, TIMP1/2, with no effect on TGF-β1 or collagen deposition. In this model of metabolic-induced damage, argatroban significantly reduced inflammation and neutrophil accumulation in the liver, globally showing early change to an anti-inflammatory, anti-fibrotic phenotype. In a recent study, Vilaseca et al. treated rats with chronic liver damage induced by CCL_4_ and TAA with rivaroxaban, an FX direct inhibitor, which reduces thrombin generation [103]. In this study, rivaroxaban reduced portal pressure and hepatic vascular resistance, confirming the amelioration of liver microcirculation. In in vitro experiments on HSC, there was no clear thrombin-related activating effect. Otherwise, rivaroxaban treatment exerted an anti-fibrotic effect on mRNA expression of αSMA, COL1A1, PDGFβ, TIMP1/2 and TGF-β1. Moreover, rivaroxaban reduced fibrin deposition and ameliorated sinusoidal architecture, as seen in TEM analysis, thus suggesting a direct effect on microthrombosis. 

In summary, preclinical studies suggest a direct anti-fibrotic effect of oral anticoagulants, which ameliorates liver microvascular perfusion, with an anti-fibrotic reprogramming on HSCs and, at last, reduced fibrin and collagen deposition. However, scant data exist on the use of direct oral coagulant in cirrhotic patients, and prescription is currently limited in this population, with few exceptions in patients with compensated disease [109]. Some registry-based studies are exploring the use of DOACs with promising results [110,111,112]; however, high-quality evidence in the form of clinical trials is eagerly awaited to confirm the safety profile of these drugs and, potentially, their impact on the natural history of the disease.

## 4. Future Directions: Hemostasis as Immune Response

The use of confocal microscopy recently shed a light on mechanisms of cell interactions in sterile or septic injury, due to the in vivo visualization allowed by the instrument [113,114]. The study of hepatic microcirculation, by in vivo visualization of sinusoids, confirmed a central role of the liver in the clearance of bacteria, as demonstrated after inoculation of S. aureus in a murine model [115,116,117]. Kupffer cells first gather in liver sinusoids after bacteria inoculation, and afterwards, neutrophils and platelets assemble and remain in the liver vasculature by VWF secretion and binding [116,118]. The platelet–neutrophil interaction leads to the organized destruction of neutrophils and the release of neutrophil extracellular traps (NETs), which are networks of neutrophil DNA and histones which entrap and kill bacteria gathered in the sinusoids [119]. This organized neutrophil death program is different from necrosis and apoptosis, and has been called NETosis [120]. While it is crucial in innate immune response, its uncontrolled activation may lead to tissue injury, and several experiments have demonstrated colocalization of NETs and subsequent necrosis. Hemostasis directly interact with NETosis by activated platelets and activation of coagulation in the site of the immune response [118,121,122]. Moreover, the demonstration of a VWF binding to histones, which precedes the discovery of NETs, suggests a continuum in hemostasis activation and tissue response to bacteria [123]. Therefore, in recent years, hemostasis has been revised as a direct effector of immune innate response, and in 2013, Engelmann used the term “immunothrombosis” to define thrombosis as an uncontrolled, deranged immune response to tissue injury [124]. Moreover, several studies have demonstrated an association between NET production and thrombosis [125,126,127,128]. Recently, McDonald et al. demonstrated an in vivo intravascular coagulation into sinusoids in response to sepsis (LPS administration and S. aureus inoculation in mice), which colocalize with NETs formation and tissue injury [129]. Interestingly, in this experiment, NET inhibition reduced thrombin activation and organ damage, while anticoagulation with argatroban alone did not reveal any effect on tissue injury. Collectively, these results confirm that the interaction between immune responses, platelets and coagulation is crucial in organ homeostasis in response to exogenous damage stimuli [122]. Therefore, immunothrombosis may represent a global mechanism which mediates tissue injury in response to acute and chronic damage and precedes fibrosis. In hepatology, increasing evidence advocate a pathogenic role for bacterial translocation from gut to general circulation [130,131]. Bacterial translocation is due to the increase of portal hypertension alongside liver disease severity, thus increasing gut permeability and disrupting the intestinal barrier [132]. This chronic exposition of enteric pathogens is associated with a progressively worsening inflammatory state, which has been recently presumed to be one of the main pathophysiological events in the development of cirrhosis-related complications [132,133,134]. Bacterial translocation is also associated with VWF, FVIII increase and platelet hyperactivation, thus confirming a pro-hemostatic role [79,135,136,137,138]. As immunothrombosis may originate from excessive response to bacteria in the liver vasculature, the existence of a chronic pathogen exposition may be crucial in sustaining inflammation, micro-thrombosis and consequent parenchymal extinction. Studies on the potential link with immune response, hemostasis activation and consequent fibrosis and disease progression are intriguing and highly anticipated. 

## 5. Conclusions

**Figure 2:**
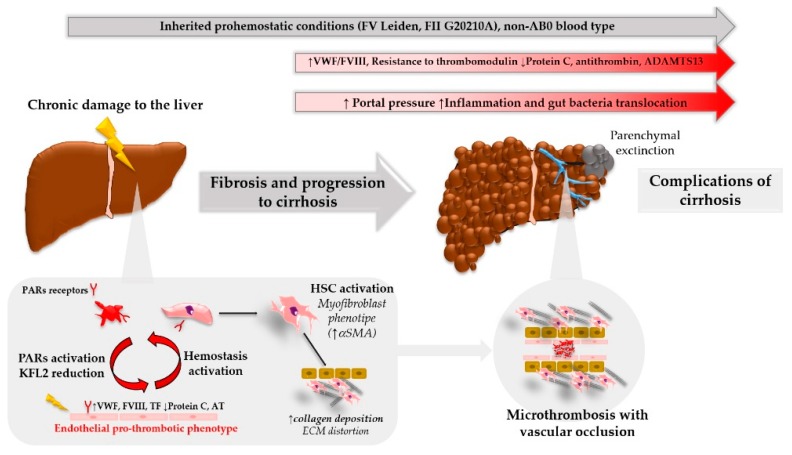
Hemostasis activation and liver disease progression.

Hemostasis has a non-negligible impact on liver fibrosis, as it induces a pro-fibrotic, activated HSC phenotype through thrombin–PARs interaction. Moreover, the increasing comprehension of liver immunology elucidates the crucial role of hemostasis in tissue injury mechanisms and may offer new potential druggable pathways by further defining this complex interplay. A pro-hemostatic milieu in liver microcirculation due to repetitive harmful stimuli may drive sinusoidal microthrombosis, which leads to parenchymal extinction and disease progression (Figure 2). As a result, an inherited or acquired pro-hemostatic imbalance is associated with fibrosis progression in pre-clinical and clinical studies. Moreover, anticoagulant drugs reduce fibrosis development, and may impact the natural history of liver disease, even in late stages of cirrhosis, which display a complex hemostatic balance. Therefore, the ever more precise understanding of the mechanisms that regulate hemostasis and its interactions with the pathophysiology of tissue damage will make it possible to better define new therapeutic targets in the clinical challenge of dampening liver fibrosis.

## Figures and Tables

**Table 1:** Studies exploring the impact of coagulation on liver fibrosis.

Reference	Experimental Model	Pathway Explored	Methods	Results
Chambers 1998 [24]	human fetal lung fibroblasts	PAR-1	Exposure to incremental dose of thrombin; TRAPs (thrombin receptor-activating peptide) +/− inhibitors (hirudin/Phe-Pro-ArgCH2CL)	Thrombin ↑ αI-procollagen mRNA through PAR-1 activation
Gaça 2002 [25]	Cultured stellate HSEC	Thrombin, tryptase/PAR 1–2	PAR 1/2 mRNA RT-PCR analysis + northern blotting in lysate of HSEC. Use of PD98059 (kinase inhibitor)	↑ PAR-1/2 while fibroblast transforms in myofibroblast phenotype ↑ HSC proliferation by PARs
Fiorucci et al. 2004 [26]	rat HSC cell line; BDL cirrhotic rat	Thrombin-PAR_s_	type I collagen mRNA expression; quantitative morphometric analysis; hepatic and urinary excretion of hydroxyproline	Thrombin triggers HSC activation and collagen deposition via PARs, prevented by PAR_1_ antagonist
J Gillibert Duplantier et al. 2007 [38]	Human hepatic myofibroblasts	PAR-1; COX-2; Akt-1; platelet derived growth factor (PDGF)	Cell migration; RNA isolation and analysis for Prostaglandin E2 receptor; analysis of Akt-1 phosphorylation and PDGF-receptor phosphorylation.	Thrombin inhibits human hepatic myofibroblast migration via PAR-1; Thrombin inhibits PDGF induced migration (inhibition of PI3K)
Martinelli 2007 [35]	Patients with HCV (287 european, 90 brazilian)	PAR1	Cross-sectional study; fibrosis evaluated by liver biopsy; polymorphism of PAR-1 gene analysis (−1426 C/T, IVS-14, −506 I/D	↑ fibrosis in TT genotype of 1426 C/T polymorphism
Rullier 2008 [32]	PAR-1 ^−/−^ and +/− mice exposed to CCL_4_	PAR1	Histology; RT-PCR for type I collagen, MMP-2, PDGFβ-r, MP-1, mRNA	↓ fibrosis and activated fibrogenic cells ↓ type I collagen, MMP-2, PDGFβ-r mRNA ↓ T lymphoctyes infiltration
B. P. Sullivan et al. 2010 [33]	Bile duct epithelial cells (BDECs); PAR1^−/−^, TF +/−, mice with low levels of human TF expression. All mice were fed with BDEC toxicant (ANIT); Human Liver Samples from patients with PBC/PSC	TF, PAR-1, αVβ6	Real-Time PCR of snap-frozen liver or adherent cells; immunofluorescence on liver frozen sections for αVβ6	TF and PAR-1 deficiency ↓ Liver Fibrosis/αVβ6 mRNA ↑ TGF-β1 related αVβ6 expression by PAR-1 αVβ6 inhibition ↓ fibrosis ↑ TF and PAR-1 mRNAs in livers from PBC/PSC patients
V. Knight et al. 2012 [31]	HSC cells; HSEC cells; (PAR-2 knockout mice; C57BL/6 mice; CCl_4_ cirrhotic mice	PARs	Hepatic hydroxyproline content in frozen liver tissue; PCR analysis of MMP-2, TIMP-1 and PAR-1/2; identification of α-SMA, F4/80 and CD68; TGF-β1 Production In Vitro; HSC Proliferation in Response to PAR Activation; Hepatic TGF-β1 Content	PAR-2 Deficiency ↓ Fibrosis/ procollagen mRNA/Hydroxyproline Content/ Stellate Cell Activation/ Hepatic TGF-β1 Expression/MMPs/ Activated Hepatic Macrophages; PAR 1/2 ↑ HSC Collagen Production/TGF-β1
R. Nault et al. 2016 [34]	PAR-1 ^−/−^ and +/− mice exposed to to TCDD (progression to NASH)	PAR-1;	Identification of Fibrin(ogen)	TCDD Exposure Activates the Coagulation Cascade; ↓ inflammation and collagen deposition in PAR-1 ^−/−^
V. Knight et al. 2017 [30]	PAR-1 ^−/−^ mice; HSC cells; CCl_4_ treated mice	TF and PARs	Hepatic fibrosis assessment; Hepatic collagen content; Gene expression of TGF-β1, MMP-2, TIMP 1, PAR1 and 2; expression TGF-β1	↓ fibrosis/MMP2/activated macrophages in TF and PAR-1 ^−/−^

**Table 2:** Main studies exploring anticoagulant-antifibrotic strategies.

Reference	Drug	Animal Model	Fibrosis/Cirrhosis Induction	Fibrosis Assesment	Results
Duplantier 2004 [28]	Wistars rat	Thrombin antagonist SSR182289	CCL_4_ (three or seven week exposure)	Histology; immunohistochemistry (IHC) for αSMA collagen type I, MMP-2, TIMP-1, and TIMP-2 mRNAs by RT-PCR	↓ 30% fibrosis (7 week CCL_4_ exposure_)_ Early ↓αSMA positive cells/TIMP-1 mRNA
Abe 2007 [99]	Dalteparin	Female Wistars Rats	CCL_4_	Histology; IHC	↓ fibrosis, ↑HGF ↓TGF-β1, COL1A1, αSMA ↓ PDGF induced HSC proliferation
Anstee 2008 [101]	Warfarin	FV Leiden mutant mice, C57BL/6 control animals anticoagulated mice	CCL_4_	Histology; Liver Hidroxiproline content; αSMA mRNA expression	↑ fibrosis 80% in male FV mutant Warfarin effect: ↓ Hidroxiproline content ↓ fibrosis scores Effect blunted in FV mutant
Kassel 2012 [102]	Argatroban (via micro-osmotic pump)	LDLr^−/−^ mice	Western diet	Histology); real time PCR hepatic mRNA expression of αSMA, COL1A1, PDGFβ, TIMP1/2, TGF-β1; IHC (anti CD68, F4/80, αSMA); MCP-1 Elisa	No change in collagen deposition ↓ αSMA, COL1A1, PDGFβ, TIMP1/2 No ↓TGF-β1 ↓inflammation (↓neutrophil/macrophage accumulation)
Cerini 2016 [100]	Enoxaparin	Male Wistars Rats	CCL_4_ (acute vs short vs long term exposure); TAA	Histology; IHC (anti FBN/αSMA/CD68); expression of procollagen I/ αSMA on isolated HSC	↓25–26% in short and long term CCL_4_ exposure; ↓ 41% in TAA ↓PP and HVR ↓αSMA, procollagen I in HSC No change on inflammation
Vilaseca 2017 [103]	Rivaroxaban	Cirrhotic wistar rats	CCL_4_; TAA	Histology; TEM analysis; Liver Hidroxiproline content; IHC (anti fibrinogen/αSMA/CD68) and IF (anti FBN, anti VWF); real time PCR hepatic mRNA expression of αSMA, COL1A1, PDGFβ, TIMP1/2, TGF-β1; in vitro thrombin action on HSC	No ↓in CCL_4_, ↓25% TAA improved sinusoidal architecture ↓Hidroxiproline content/collagen/fibrin deposition ↓PP and HVR ↓HSC activity of profibrotic genes ↓VWF expression in vasculature No direct activity on HSC (in vitro studies)
Li 2017 [98]	Aspirin (low/high dose), enoxaparin	Sprague-Dawley rats	TAA	Histology (METAVIR score)	↓ in all treatment group (> for high dose aspirin)
